# Bio-physiological susceptibility of the brain, heart, and lungs to systemic ischemia reperfusion and hyperoxia-induced injury in post-cardiac arrest rats

**DOI:** 10.1038/s41598-023-30120-1

**Published:** 2023-02-28

**Authors:** Tomoaki Aoki, Vanessa Wong, Yusuke Endo, Kei Hayashida, Ryosuke Takegawa, Yu Okuma, Muhammad Shoaib, Santiago J. Miyara, Tai Yin, Lance B. Becker, Koichiro Shinozaki

**Affiliations:** 1 Feinstein Institutes for Medical Research, Northwell Health, Manhasset, NY USA; 2 Department of Neurosurgery, Sonoda Daiichi Hospital, Tokyo, Japan; 3 Department of Emergency Medicine, Donald and Barbara Zucker School of Medicine at Hofstra/Northwell Health, Hempstead, NY USA

**Keywords:** Experimental models of disease, Translational research, Outcomes research

## Abstract

Cardiac arrest (CA) patients suffer from systemic ischemia–reperfusion (IR) injury leading to multiple organ failure; however, few studies have focused on tissue-specific pathophysiological responses to IR-induced oxidative stress. Herein, we investigated biological and physiological parameters of the brain and heart, and we particularly focused on the lung dysfunction that has not been well studied to date. We aimed to understand tissue-specific susceptibility to oxidative stress and tested how oxygen concentrations in the post-resuscitation setting would affect outcomes. Rats were resuscitated from 10 min of asphyxia CA. Mechanical ventilation was initiated at the beginning of cardiopulmonary resuscitation. We examined animals with or without CA, and those were further divided into the animals exposed to 100% oxygen (CA_Hypero) or those with 30% oxygen (CA_Normo) for 2 h after resuscitation. Biological and physiological parameters of the brain, heart, and lungs were assessed. The brain and lung functions were decreased after CA and resuscitation indicated by worse modified neurological score as compared to baseline (222 ± 33 vs. 500 ± 0, *P* < 0.05), and decreased PaO2 (20 min after resuscitation: 113 ± 9 vs. baseline: 128 ± 9 mmHg, *P* < 0.05) and increased airway pressure (2 h: 10.3 ± 0.3 vs. baseline: 8.1 ± 0.2 mmHg, *P* < 0.001), whereas the heart function measured by echocardiography did not show significant differences compared before and after CA (ejection fraction, 24 h: 77.9 ± 3.3% vs. baseline: 82.2 ± 1.9%, *P* = 0.2886; fractional shortening, 24 h: 42.9 ± 3.1% vs. baseline: 45.7 ± 1.9%, *P* = 0.4658). Likewise, increases of superoxide production in the brain and lungs were remarkable, while those in the heart were moderate. mRNA gene expression analysis revealed that CA_Hypero group had increases in *Il1b* as compared to CA_Normo group significantly in the brain (*P* < 0.01) and lungs (*P* < 0.001) but not the heart (*P* = 0.4848). Similarly, hyperoxia-induced increases in other inflammatory and apoptotic mRNA gene expression were observed in the brain, whereas no differences were found in the heart. Upon systemic IR injury initiated by asphyxia CA, hyperoxia-induced injury exacerbated inflammation/apoptosis signals in the brain and lungs but might not affect the heart. Hyperoxia following asphyxia CA is more damaging to the brain and lungs but not the heart.

## Introduction

Cardiac arrest (CA) is a major cardiovascular health issue afflicting approximately 600,000 patients each year in the United States^1^. Post-CA syndrome is a lethal condition characterized by high mortality and systemic ischemia–reperfusion (IR) injury resulting in multiple organ damage, of which the most susceptible is the central nervous system^2,3^. However, little is known about tissue-specific patterns of damage after CA. Furthermore, most CA patients are treated with supplemental oxygen without a clear understanding of the optimal dose for recovery. Sustained hyperoxia can induce toxic injury that worsens mortality and organ dysfunction of CA patients^4,5^. Studying the tissue-specific pattern of damage and its association with hyperoxia-induced injury are important to better understand the pathophysiology of post-CA syndrome and to improve survival after CA.

We have recently reported that post-resuscitation normoxic therapy mitigates oxidative stress in multiple organ systems and attenuates post-CA organ injury in a rat asphyxia CA model^6–8^. Our data revealed that hyperoxia-induced oxidative stress affected proteins and deoxyribonucleic acid (DNA) in the brain, lungs, and kidneys after CA with increases in carbonyl protein and 8-hydroxy-2’-deoxyguanosine (8OHdG) levels, respectively. However, the heart did not show significant increases in these oxidative stress indicators, suggesting that there is a tissue-specific susceptibility to systemic IR injury and hyperoxia-induced oxidative stress. Although we have shown tissue-specific metabolic profiles after prolonged CA^9^, most studies in resuscitation science have only focused on a damage in the central nervous system^10–12^. Several studies have demonstrated information on injuries of other organs such as the heart, lungs, and kidneys.

Byrne et al. reported out-of-hospital CA (OHCA) survivors had significantly increased five-year risks of stroke, atrial flutter (AF), acute coronary syndrome (ACS), and heart failure (HF)^13^. Moreover, Tsai et al. reported myocardial injury after CA in a rat model of ventricular fibrillation CA^14^. Although, lung and kidney injuries after CA have been reported in clinical studies^15,16^ and experimental animal models^17–19^, the data are scarce and few studies have reported results from simultaneous comparison among these tissues after CA. Moreover, Mai et al. have recently opened a new concept, “lung-brain coupling^20,21^”, highlighting the lung’s role in modulating the response to post-CA organ damage. Therefore, it is noteworthy to see a link between oxidative stress and the organ damage and to test how oxygen concentrations in the post-resuscitation setting affects outcomes particularly on the brain and lungs.

Herein, we evaluated biological and physiological parameters of the brain, heart, and lungs, with detailed mRNA expression profiles of inflammation and apoptosis related genes in each of these tissues. We examined animals with or without CA, and those were further divided into the normoxia or hyperoxia group in order to understand the tissue-specific susceptibility to systemic IR injury and its association with concomitant hyperoxia-induced injury.

## Methods

The Institutional Animal Care and Use Committees of Feinstein Institutes for Medical Research approved the animal protocol (#2016–004). All methods were performed in accordance with the National Institutes of Health Guide for the Care and Use of Laboratory Animals and ARRIVE guidelines.

### Animal preparation

**Figure 1:**
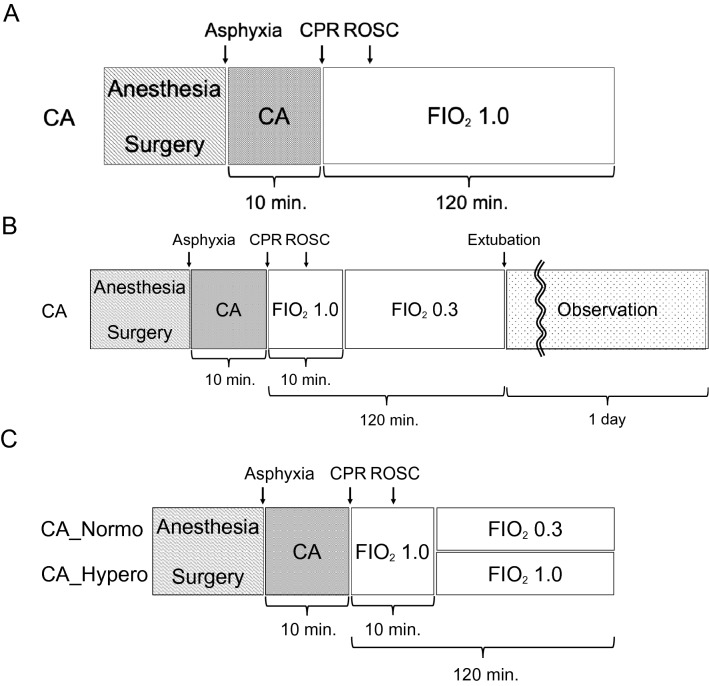
Animal protocols for each study. (**A**) Animal protocol for dihydroethidium (DHE) assay. Tissues of rats 120 min after successful resuscitation from a 10-min asphyxia arrest treated with inhaled 100% O_2_ were collected and applied to cryosection, followed by DHE staining. DHE derived 2-hydroxyethidium (2-OH-E^+^) fluorescence was compared between CA group and non-interventional control rat tissues. (**B**) Animal protocol for physiological assessment. Rats 120 min after successful resuscitation from a 10 min asphyxia arrest treated with inhaled 30% O_2_ were extubated and observed until 24 h after CPR. For the assessment of brain function, modified neurological deficient score (mNDS) was evaluated. mNDS before anesthesia (Baseline) and 24 h after CA were compared. For the assessment of heart function, echocardiography was performed to compare ejection fractioning (EF) and fractional shortening (FS) between baseline and 24 h after CA. For the assessment of lung injury, lung wet to dry (W/D) weight ratio was measured. Wet lung weight was measured just after extraction, and dry lung weight was measured after drying over for 1 week in a 37 ℃ oven. Non-interventional control rat lung and 24 h after CA rat lung were compared. (**C**) Animal protocol for real time PCR. Similar with the protocols above, rats were assigned into 2 groups: (1) 120 min after successful resuscitation from 10 min asphyxia arrest treated with inhaled 30% O_2_ (CA_Normo) and (2) those with inhaled 100% O_2_ (CA_Hypero). In CA_Normo group, 10 min after CPR inhaled oxygen concentration was changed to 30%, whereas inhaled oxygen concentration was kept 100% in CA_Hypero group. At 2 h after CPR, the brain, heart, and lung were collected and mRNA extraction, followed by cDNA synthesis and real-time PCR were performed. Additionally, tissues from non-interventional control rat group were also collected for reference of mRNA gene expression.

We performed all instrumentation according to the previously described protocol^7^ (Fig. 1A). In brief, adult male 12–16-week-old Sprague–Dawley rats (400–550 g, Charles River Laboratories, Wilmington, MA) were anesthetized with 4% isoflurane (Isosthesia, Butler-Schein AHS, Dublin, OH) and intubated with a 14-gauge plastic catheter (Surflo, Terumo Medical Corporation, Tokyo, Japan). We used only male rats to reduce potential variation among animals that may cause confounding outcomes from hormonal and/or genetic differences. Rats were mechanically ventilated (Ventilator Model 683, Harvard Apparatus, Holliston, MA) at a minute ventilation (MV) volume of 180 ml per minute, a respiratory rate of 45 breaths per minute. In this study, we used 1 ventilation setting for all animals at all times and did not change the MV or respiratory rate over the experiments. Anesthesia was maintained with isoflurane at 2% and a fraction of inspired oxygen (FIO_2_) of 0.3. Local analgesia with subcutaneous injection of lidocaine (Fresenius Kabi, Lake Zurich, IL) and bupivacaine (Fresenius Kabi, Lake Zurich, IL) was administered at the left thigh and lower middle abdomen prior to make an incision. Core (esophageal) temperature was maintained at 36.5 ± 1.0 ℃ during the surgical procedure. After instrumentation, neuromuscular blockade was achieved by slow intravenous administration of 2 mg/kg of vecuronium bromide (Hospira, Lake Forest, IL) and asphyxia was induced by turning off the ventilator. CA normally occurred 3 to 4 min after asphyxia started. The CA group received chest compression cardiopulmonary resuscitation (CPR) after a 10-min asphyxia CA. We defined CA as a mean arterial pressure of < 20 mmHg; CA was completely untreated during this initial 10-min interval. After the 10-min asphyxia, mechanical ventilation was restarted at an FIO_2_ of 1.0 and manual chest compression CPR was delivered simultaneously. Chest compressions were performed with 2 fingers over the sternum at a rate of 260 to 300 per minute. At 30 s after beginning of CPR, a 20 µg/kg bolus of epinephrine was given to rats through a venous catheter. Following return of spontaneous circulation (ROSC), defined as mean arterial pressure > 60 mmHg, CPR was discontinued. If ROSC did not occur by 5 min of CPR, resuscitation was terminated. Mechanical ventilation was discontinued at 2 h after CPR, after which rats were euthanized, and tissues were collected for subsequent analysis unless otherwise described below.

### Organ damage assessment for the brain, heart, and lungs

Animals received aforementioned procedures including surgical preparation, drug administrations, and CPR (Fig. 1B). At 10 min after CPR, FIO_2_ was switched back to 0.3. Mechanical ventilation was discontinued at 120 min after CPR and rats were monitored up to 24 h. Immediately after extubating, a subcutaneous injection of 0.65 mg/kg buprenorphine (Ethiqa XR, Fidelis Pharmaceuticals, North Brunswick Township, NJ) was given to the animals for analgesia. Eight rats were subject to this study and 1 rat died within 24 h, and therefore the rest of the 7 rats were subjected to further analysis. Modified neurological deficit score (mNDS) and echocardiography were compared between baseline and 24 h after CPR for each rat. Survived rats received echocardiography to measure ejection fraction (EF) and fractional shortening (FS). After assessment of echocardiography, rats were euthanized and tissues were collected for subsequent analysis including wet/dry ratio of the lung. Postsurgical care including animal housing and observation were provided by the animal facility.

### Modified neurological deficit score

At 24 h after CPR in the physiological evaluation study, survived rats were subject to mNDS assessment by a single researcher^22^. The score (0–500 points, worst to best) is composed of 5 parameters: general appearance (0–200 points), cranial nerve (0–100 points), motor (0–50 points), sensory (0–50 points), and coordination (0–100 points). The sub-parameter of coordination skill was composed of four kinds of tests, namely righting reflex test, placing reflex test, stopping at edge of table test, and ledge traverse test.

### Echocardiography

Trans-thoracic echocardiography for myocardial function assessment was performed pre- and 24 h after CA by a single investigator using standard echocardiographic methods^23,24^. At both time points, rats were anesthetized with inhaled 2% isoflurane without intubation, and examined in the supine position after the chest fur was shaved. 2D and M-mode measurements were applied with a CX50 ultrasound machine (S12-4 sector array transducer; Philips, Amsterdam, Netherlands) by using a 12–4 MHz probe. Views were taken after optimization of gain, angulation, and rotation. M-mode measurements were performed at or just below the level of the heart papillary muscles. Ultrasound measurements included EF and FS calculated as follows: EF = ((left ventricular end-diastolic volume) − (left ventricular end-systolic volume)) ÷ left ventricular end-diastolic volume × 100 (%); FS = ((left ventricular end-diastolic diameter) − (left ventricular end-systolic diameter)) ÷ left ventricular end-diastolic diameter × 100 (%).

### Wet/dry weight ratio of the lung

After the assessment of mNDS and echocardiography, rats were euthanized and the left lung of each rat was weighed immediately after collection and then placed into a 37 °C oven for 1 week. After drying, the left lung was weighed again to determine a lung wet/dry (W/D) ratio. The left lung in the non-interventional control rat group was also collected and W/D ratio weight was calculated for comparison.

### Fluorescent dihydroethidium assay for superoxide detection

Dihydroethidium (DHE, Sigma-Aldrich Corp., St. Louis, MO) staining was applied to measure superoxide production according to a previously reported protocol^25^. Briefly, at 2 h after CPR, anesthesia was re-induced in rats with 2% isoflurane and rats were perfused transcardially from left ventricle with 50 ml 4 ℃ saline. Immediately after perfusion, brain, heart, and lung tissues were collected. At this time, tissues of control rats were obtained simultaneously. For DHE staining, small pieces of collected heart and lung tissues were mounted into molds filled with O.C.T. compound (Fisher Healthcare, Waltham, MA), and the molds were immediately frozen above liquid nitrogen. The frozen molds were maintained at − 20 ℃ over 1 h and sliced into 10–15 μm thick sections by cryostat. Dried tissue sections were stained by soaking the slide glasses into a staining solution containing 5 μM DHE, 0.05 mg/ml DNA sodium salt from salmon testes (Sigma-Aldrich Corp., St. Louis, MO), and 50 μM diphenyleneiodonium chloride (DPI, Thermo Scientific, Waltham, MA). The fluorescence histological samples were mounted with VECTORSHIELD hard set mounting medium with 4, 6-Diamido-2-Phenylindole (DAPI) (Vector Laboratories, Inc., Burlingame, CA) followed by the visualization of DHE derived 2-hydroxyethidium (2-OH-E^+^), a specific adduct of cellular O_2_^•−^, and DAPI by using LSM 880 confocal microscopy imaging system (Carl Zeiss, Inc., Jena, Germany). Acquired fluorescence images were analyzed with Fiji/ImageJ software (http://rsbweb.nih.gov/ij/, Version; 2.0.0-rc-69/1.52p, Rasband, W.S., ImageJ, U.S. National Institute of Health, Bethesda, MD) to assess fluorescence intensity of the 2-OH-E^+^ signals. To quantify the degree of superoxide production, we used a microplate reader to measure the fluorescence intensity. The brain, heart, and lung homogenates were immediately treated with lysate containing protease inhibitors (cOmplete™, Mini Protease Inhibitor Cocktail, Sigma-Aldrich Corp., St. Louis, MO) after tissue collections. Samples were then applied to 96 well plate with 10 μM DHE, 0.1 mg/ml DNA sodium salt from salmon testes and 100 μM DPI. Relative fluorescence intensity of 2-OH-E^+^ in each experimental tissue compared to that from naïve rats was measured by the fluorescence microplate reader (excitation wavelength; 485 nm, emission wavelength; 570 nm, Infinite®200 PRO, TECAN, Männedorf, Switzerland). Multiple well measurements for the brain, heart, and lung homogenates were performed.

### S100B

S100B concentrations in the plasma collected at baseline, 30 min, 1 h, and 2 h after CA were measured by using a rat S100B ELISA kit (MyBioSource, MBS2021416, San Diego, CA), as per manufacturer’s instructions. Briefly, after preparation of reagents, plasma samples were diluted with 1 × PBS. Samples and standards were applied to the supplied 96-well microplates and incubated at 37 ℃ for 1 h. After antigen–antibody reaction, TMB Substrate solution was added. After adding Stop Solution, absorbance was detected at 450 nm using the plate reader. Finally, S100B concentration of each sample was calculated according to the standard curve.

### Troponin I

Troponin I concentrations in the plasma were measured at baseline, 30 min, 1 h, and 2 h after CA by using a rat cardiac troponin I ELISA kit (abcam, ab246529, Cambridge, United Kingdom), as per manufacturer’s instructions. Our standardized ELISA protocol was as described above.

### Real-time PCR

The same surgical procedures were performed as described above and rats were assigned into 2 groups at 10 min after CPR (Fig. 1C): (1) The post-resuscitation normoxic therapy group (*n* = 6) included those that were successfully resuscitated from 10-min asphyxia CA and treated with inhaled 30% oxygen (CA-Normo) following the initial 10 min of 100% oxygen, and (2) The post-resuscitation hyperoxia group (*n* = 6) included those treated with inhaled 100% oxygen (CA-Hypero) during the entire observational period. For all rats, the brain and heart tissues were collected at 2 h after CPR for mRNA extraction, followed by complementary DNA (cDNA) synthesis and real-time PCR. Additionally, tissues of control (naive) rats were collected to create a reference for mRNA gene expression.

RNA isolation, reverse transcription, and real-time PCR analysis for the brain, heart, and lung samples extracted at 2 h after CA were performed according to manufacturer instructions. Briefly, total RNAs were extracted and reverse transcribed using TRIzol Reagent® (Invitrogen, Carlsbad, CA) and SuperScript™ IV VILO™ Master Mix with ezDNase™ Enzyme (Invitrogen, Carlsbad, CA), respectively. Real-time PCR was performed using TaqMan™ Fast Advanced Master Mix (Applied Biosystems™, Waltham, MA) on the LightCycler 480 system (Roche Diagnostics, Mannheim, Germany). All primers were purchased from Thermofisher: *Glyceraldehyde-3-phosphate dehydrogenase* (*Gapdh*, TaqMan Assay ID: Rn01775763_g1), *Interleukin-1 beta* (*Il1b*, Rn00580432_m1), *Interleukin-6* (*Il6*, Rn01410330_m1), *Transforming growth factor-beta 1* (*Tgfb1*, Rn00572010_m1), *Intercellular adhesion molecule-1* (*Icam1*, Rn00564227_m1), *Nuclear factor-kappa beta 1* (*Nfkb1*, Rn01399583_m1), *Tumor necrosis factor (Tnf) receptor-associated factor-6* (*Traf6*, Rn01512911_m1), *Caspase-3* (*Casp3*, Rn00563902_m1), *Caspase-9* (*Casp9*, Rn00581212_m1), *Epidermal growth factor* (*Egf*, Rn00563336_m1), and *B-cell leukemia/lymphoma-2* (*Bcl2*) *associated X protein* (*Bax*, Rn02532082_g1).

### Statistical analysis

Data are shown as the means with standard error of measurement (SEM) for continuous variables. For multi-group comparisons, one-way ANOVA with post-hoc analysis, Wilcoxon matched-pairs signed rank test, or Mann–Whitney test were used. Two-tailed *P* values were calculated, and *P* < 0.05 was considered statistically significant. Prism 9.1.0 (GraphPad, San Diego, CA) were used for statistical analyses.

## Results

### Superoxide production in multiple organ systems after cardiac arrest measured by fluorescent dye

**Figure 2:**
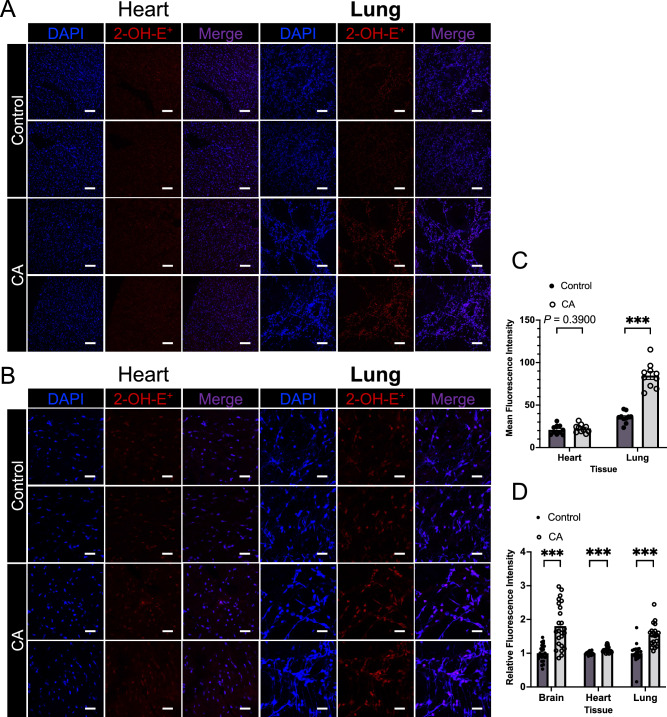
Superoxide production in tissues after cardiac arrest detected by DHE staining and fluorescence microplate reader. (**A**) Representative low-power field images of DHE staining in heart and lung. The left 3 columns are the images of heart and right 3 columns are those of lung. Upper 2 and lower 2 panels show control group and cardiac arrest group, respectively. Blue fluorescence as DAPI, red fluorescence as 2-hydroxyethidium (2-OH-E^+^), and merged panels are shown for each image. Scale bars indicate 100 µm. (**B**) Representative high-power field images of DHE staining. Scale bars indicate 25 µm. (**C**) Mean fluorescence intensity of 2-OH-E^+^ in heart and lung. Ten low-power field images of each groups are analyzed and data are shown as mean ± SEM with scatter dot plot. *** *P* < 0.001, Mann–Whitney test. (**D**) Relative fluorescence intensity of 2-OH-E^+^ in brain, heart, and lung were detected on fluorescence microplate reader (excitation wavelength; 485 nm, emission wavelength; 570 nm). Multiple well measurement of 7 paired protein samples from tissue homogenate in brain and 5 paired samples in heart, lung was applied and data were shown as mean ± SEM with scatter dot plot. ***P < 0.001, Mann–Whitney test.

DHE was used to identify superoxide in tissues obtained after CA and resuscitation^25^. DHE permeates the cell membrane, can be oxidized by intracellular superoxide, and produce fluorescent 2-OH-E^+^, which reacts specifically to superoxide and can be identified by a fluorescence microscope or microplate reader^26^. In our rat asphyxia CA model, superoxide production in tissues, after CA, were identified (Fig. 2). Fluorescent histology demonstrated higher 2-OH-E^+^ fluorescence in the CA group as compared to the control (naive) group in the lung, while no significant increase in 2-OH-E^+^ fluorescence was observed in the heart. Likewise, although there were significant increases of 2-OH-E^+^ fluorescence in all tissues (brain, heart, and lung) identified by the microplate reader, the increase was moderate in the heart. These data indicate tissue-specific patterns of superoxide production after asphyxia CA and resuscitation.

### The brain and lungs were more susceptible to asphyxia-induced CA as compared to the heart

**Figure 3:**
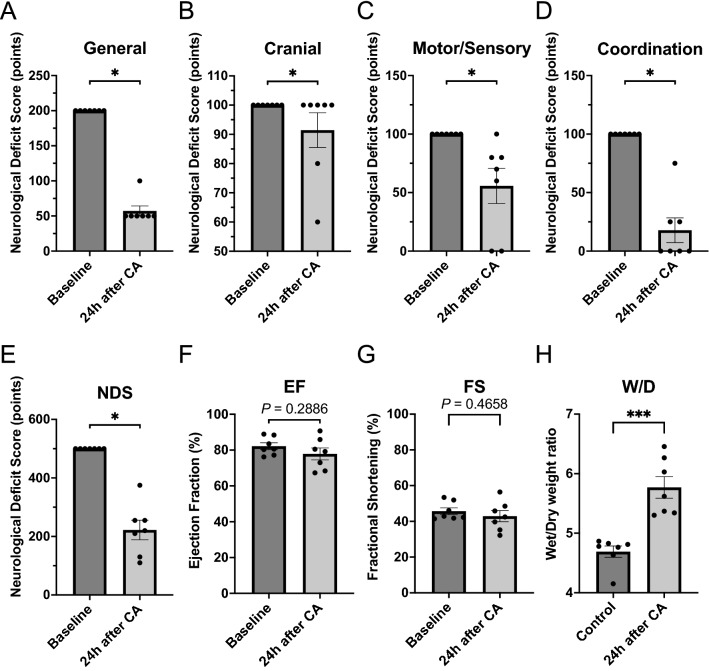
Physiological insults and functional deficits of tissues after CA. (**A**) Modified Neurological Deficit Score (mNDS), scores of general appearance. Baseline vs. 24 h after CA, N = 7. (**B**) mNDS, scores of cranial nerves. Baseline vs. 24 h after CA, N = 7. (**C**) mNDS, scores of motor/sensory nerves. Baseline vs. 24 h after CA, N = 7. (**D**) mNDS, scores of coordination. Baseline vs. 24 h after CA, N = 7. (**E**) mNDS, total scores. Baseline vs. 24 h after CA, N = 7. (**F**) Ejection fractioning (EF). Baseline vs. 24 h after CA, N = 7. (**G**) Fractional shortening (FS, %). Baseline vs. 24 h after CA, N = 7. (**H**) Wet/dry (W/D) weight ratio of lung tissue. Control vs. 24 h after CA. N = 7, respectively. Data were shown as mean ± SEM with scatter dot plot. *P < 0.05, ***P < 0.001, A–G; Wilcoxon matched-pairs signed rank test, H; Mann–Whitney test.

Figure 3 shows physiological and functional deficits of organs after CA and resuscitation. For brain function, all composing parameters of mNDS were significantly lower 24 h after CA leading to worse overall mNDS (222 ± 33) as compared to their baseline values (Fig. 3A–E). During post-resuscitation period, airway pressure significantly increased both at 20 min and 2 h after CA (Supplemental Table S1: baseline, 8.1 ± 0.2 mmHg; 20 min, 9.7 ± 0.7 mmHg; 2 h, 10.3 ± 0.3 mmHg, *P* values < 0.05 and < 0.001, respectively), furthermore lung W/D ratio, which represents the swelling and damage level of the lung, also significantly increased 24 h after CA (Fig. 3H, control: 4.7 ± 0.1; 24 h: 5.8 ± 0.2, *P* < 0.001). Contrarily, EF and FS as representative parameters of the heart function did not show significant differences between baseline and 24 h after CA (Fig. 3F, G, EF: baseline, 82.2 ± 1.9%; 24 h, 77.9 ± 3.3%, *P* = 0.2886 and FS: baseline, 45.7 ± 1.9%; 24 h: 42.9 ± 3.1%, *P* = 0.4658). These data suggest that asphyxia CA and resuscitation as a systemic IR injury might not cause physiological damage to the heart as compared to the brain and lung.

### S100B in plasma after asphyxia-induced cardiac arrest

**Figure 4:**
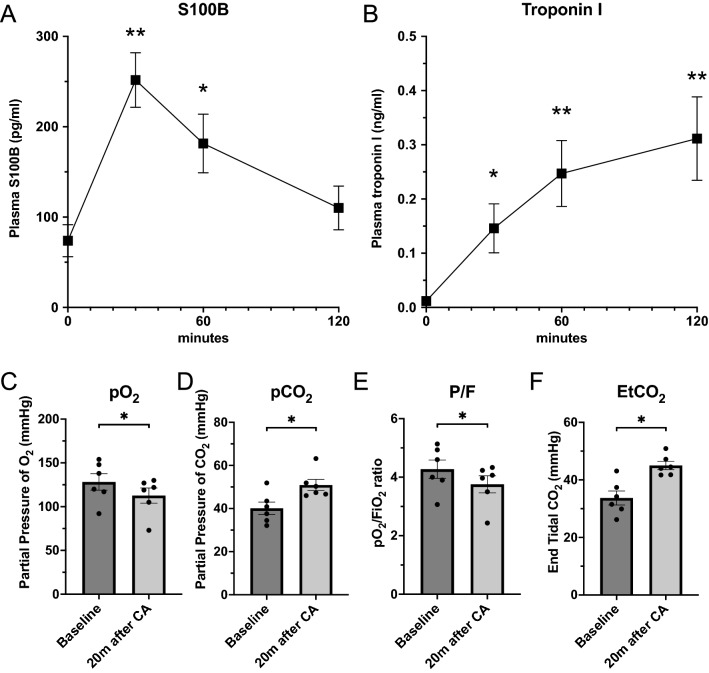
Plasma S100B concentration, plasma troponin I concentration, arterial blood gas analysis, and EtCO_2_ after CA. (**A**) Plasma S100B concentrations were detected using rat S100B ELISA kit (MBS2021416). Baseline, 30 min, 1 h, and 2 h plasma samples of 8 rats, after CA were applied to measurement. S100B levels significantly increased at 30 min and 1 h, but not at 2 h after CA and resuscitation (baseline; 73.8 ± 17.7 pg/ml, 30 min; 251.8 ± 30.2 pg/ml, 1 h; 181.5 ± 32.4 pg/ml, 2 h; 110.1 ± 24.2 pg/ml). (**B**) Plasma troponin I concentrations were detected using rat cardiac troponin I ELISA kit (ab246529). Baseline, 30 min, 1 h, and 2 h plasma samples of 8 rats, after CA were applied to measurement. Troponin I levels significantly increased gradually over time after CA and resuscitation (baseline; 0.010 ± 0.004 ng/ml, 30 min; 0.146 ± 0.048 ng/ml, 1 h; 0.247 ± 0.065 ng/ml, 2 h; 0.312 ± 0.082 ng/ml). (**C–F**) Arterial blood gas analysis was performed using iSTAT. Data at baseline, and 20 min after CA were compared. (**C**) pO_2_ was significantly decreased at 20 min after CA. (**D**) pCO_2_ was significantly increased at 20 min after CA. (**E**) pO_2_/FIO_2_ (P/F) ratio was calculated from pO_2_ and FIO_2_. P/F ratio was significantly decreased at 20 min after CA. (**F**) End tidal CO_2_ (EtCO_2_) was measured throughout the procedure and significantly increased at 20 min after CA. N = 6. Data were shown as mean ± SEM. *P < 0.05, **P < 0.01, t-test.

Figure 4A shows the time course data of S100B concentrations in the plasma. Plasma samples collected at baseline, 30 min, 1 h, and 2 h after CA revealed that S100B concentration significantly increase at 30 min and 1 h, but not at 2 h (baseline; 73.8 ± 17.7 pg/ml, 30 min; 251.8 ± 30.2 pg/ml, 1 h; 181.5 ± 32.4 pg/ml, 2 h; 110.1 ± 24.2 pg/ml), suggesting that active neural distress might occur in the acute phase after asphyxia CA.

### Arterial blood gas analysis demonstrated impaired gas exchange function of the lung after asphyxia-induced cardiac arrest

Figure 4C–F shows arterial blood gas analysis measured at baseline and 20 min after CA. pO_2_ was significantly decreased (baseline, 128.2 ± 9.4 mmHg; 20 min, 112.7 ± 8.7 mmHg, *P* < 0.05) while pCO_2_ was significantly increased after CA (baseline, 40.1 ± 2.9 mmHg; 20 min, 50.9 ± 2.6 mmHg, *P* < 0.05). Likewise, pO_2_/FIO_2_ (P/F) ratio was significantly decreased (baseline, 4.3 ± 0.3; 20 min, 3.8 ± 0.3 mmHg, *P* < 0.05) and end tidal CO_2_ (EtCO_2_) was significantly increased after CA (baseline, 33.7 ± 2.4 mmHg; 20 min, 45.0 ± 1.4 mmHg, *P* < 0.05). Collectively, gas exchange was significantly impaired by asphyxia-induced CA and resuscitation.

### Troponin I levels in plasma after asphyxia-induced cardiac arrest

Figure 4B shows the time course data of troponin I concentrations in the plasma. Plasma samples collected at baseline, 30 min, 1 h, and 2 h after CA demonstrated a gradual increase in troponin I levels over time. Troponin I concentration significantly increased after CA and resuscitation (baseline; 0.010 ± 0.004 ng/ml, 30 min; 0.146 ± 0.048 ng/ml, 1 h; 0.247 ± 0.065 ng/ml, 2 h; 0.312 ± 0.082 ng/ml), indicating that this biomarker is sensitive enough to identify minimal damage in myocardium that was not presented as physiological or functional deficits as described above.

### Realtime PCR revealed that normoxic therapy mitigated inflammatory and apoptosis related mRNA gene expression in the brain, and lung but not in the heart

**Figure 5:**
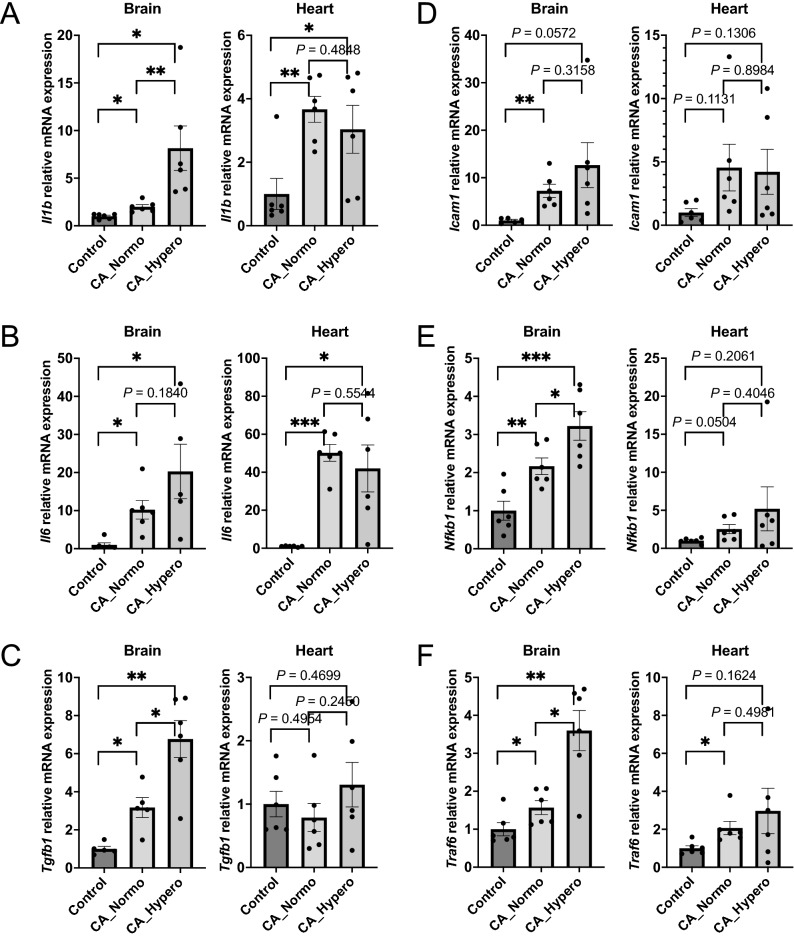
Inflammatory mRNA relative gene expressions. *Il1b, Il6, Tgfb1, Icam1, Nfkb1*, *Traf6*. Real time PCR was performed to compare mRNA gene expression. (**A**) *Il1b* was upregulated after CA in brain, which was significantly attenuated by normoxic therapy, whereas *Il1b* was not attenuated by normoxic therapy in heart. (**B**) *Il6* was upregulated after CA, though there was no significant difference between CA_Normo group and CA_Hypero group in brain. *Il6* also increased after CA, which was not attenuated by normoxic therapy in heart. (**C**) *Tgfb1* was upregulated in CA_Hypero group in brain, which was significantly attenuated by normoxic therapy. *Tgfb1* was not upregulated after CA in heart. (**D**) *Icam1* was upregulated after CA, though there was no significant difference between CA_Normo group and CA_Hypero group in brain. *Icam1* also tended to increase after CA, which was not attenuated by normoxic therapy in heart. (**E**) *Nfkb1* was upregulated in CA_Hypero group in brain, which was significantly attenuated by normoxic therapy. *Nfkb1* was not upregulated after CA in heart. (**F**) *Traf6* was upregulated in CA_Hypero group in brain, which was significantly attenuated by normoxic therapy. *Traf6* tended to be upregulated after CA, though there was no significant difference between CA_Normo group and CA_Hypero group in heart. N = 4–6. *P < 0.05, **P < 0.01, ***P < 0.001 t-test.

**Figure 6:**
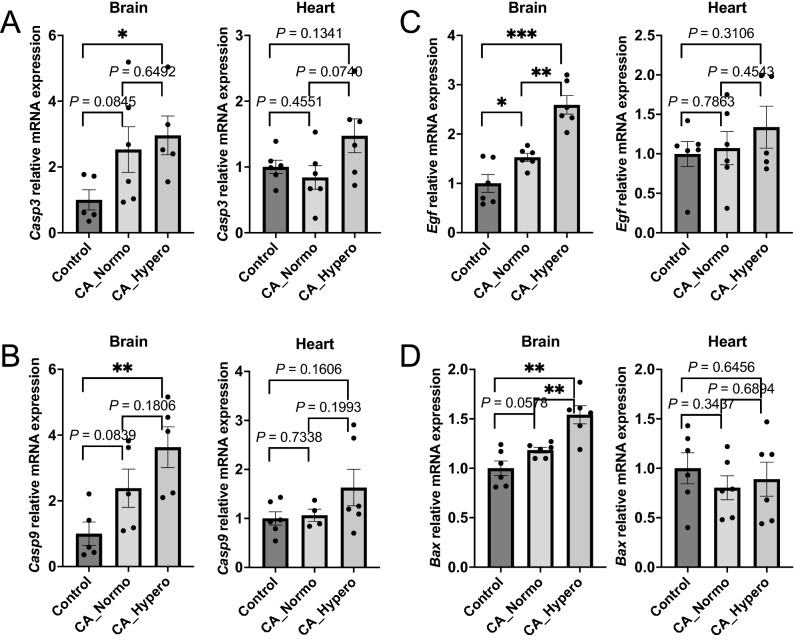
Apoptotic mRNA relative gene expression. *Casp3*, *Casp9, Egf*, *Bax*. Real time PCR was performed to compare mRNA gene expression. (**A**) *Casp3* was upregulated in CA_Hypero group but not in CA_Normo group, though there was no significant difference between the groups in brain. *Casp3* was not upregulated after CA in the heart. (**B**) *Casp9* was upregulated in CA_Hypero group but not in CA_Normo group, which tended to be attenuated by normoxic therapy in brain. *Casp9* was not upregulated after CA in heart. (**C**) *Egf* was upregulated in CA_Hypero group in brain, which was significantly attenuated by normoxic therapy. *Egf* was not upregulated after CA in heart. (**D**) *Bax* was upregulated in CA_Hypero group in brain, which was significantly attenuated by normoxic therapy. *Bax* was not upregulated after CA in heart. N = 4–6. *P < 0.05, **P < 0.01, ***P < 0.001. t-test.

Figures 5, 6 show mRNA gene expression of inflammatory and apoptosis related genes compared within the control (naive) group, CA_Normo group, and CA_Hypero group. *Il1b* was upregulated after CA in the brain, which was significantly attenuated by normoxic therapy. *Il6* was also upregulated after CA and appeared to be lowered by normoxic therapy, although it was not statistically significant between the normoxia and hyperoxia groups in the brain. Both *Il1b* and *Il6* were significantly upregulated after CA and attenuated by normoxic therapy in the lung, whereas the attenuation of upregulated *Il1b* and *Il6* by normoxic therapy was not detected in the heart, unlike the brain and lung (Fig. 5A, B, Supplemental Figure S1). Likewise, mRNA expression of other inflammatory genes, *Tgfb1*, *Icam1*, *Nfkb1*, and *Traf6*, were upregulated after CA and they appeared to be attenuated by normoxic therapy in the brain, while those were not remarkably upregulated or attenuated by the normoxic therapy in the heart (Fig. 5C–F). All of 4 apoptotic related genes such as *Casp3*, *Casp9*, *Egf*, and *Bax*, were upregulated after CA and they appeared to be attenuated by normoxic therapy in the brain, whereas no difference of apoptosis related gene expression was found between the three groups in the heart (Fig. 6). Collectively, it is inferred that increases in inflammation related mRNA gene expression in the heart might not be derived from hyperoxia-induced oxidative stress, but may rather be directly associated with the injury from CA and resuscitation. In addition, apoptosis related mRNA gene expression did not increase in the heart after CA, which is consistent with the findings described above.

## Discussion

Oxidative stress is known to induce DNA damage, protein degradation, inflammation, cellular apoptosis, and further organ injury in many diseases^27,28^. It can be inferred that an upregulation of reactive oxygen species (ROS) and/or a down-regulation of antioxidation systems may contribute to post-CA cell injuries, and reckless and sustained use of supplemental oxygen could worsen organ dysfunction. Cellular superoxide, which is one of the most important ROS, can be identified with cell membrane permeable fluorescent dyes such as DHE. Our findings are highlighted by the successful detection of superoxide levels in tissues by using our highly fidelity asphyxia-induced CA model. The findings from this study along with our previous reports support that the systemic IR injury induces protein and DNA degradations indicated by increases in carbonyl protein and 8OHdG levels, respectively^6^, and this damage process is highly associated with cellular superoxide production after prolonged CA and resuscitation. Either in the presence or the absence of hyperoxia, the immunohistochemistry staining revealed that the CA group showed severe injury with increased levels of heme oxygenase-1 and high mobility group box-1 in the brain and lung as compared to the sham-surgery group. In addition, long-term neurological scores and survival rates were investigated in the previous report, in which CA animals treated with normoxia and those with hyperoxia were subject to the same length of asphyxia time (10 min) as the present study^6^. In this previous report, the animals with normoxia demonstrated a higher survival rate (77%) at 48 h after resuscitation as compared to those with hyperoxia (28%, *P* = 0.010). Along with improved survival rates, the normoxia group had significantly lower neurological deficit score (359 ± 140, *500–0 points, worst to best in the previous study) as compared to the hyperoxia group (452 ± 85, *P* = 0.026). These results were further supported by our current findings of increases in mRNA gene expression of inflammatory cytokines and their association with hyperoxia-induced injury in the brain.

CA and resuscitation lead to post-CA syndrome, which is characterized by systemic IR injury, anoxic brain damage, and post-arrest myocardial dysfunction^29,30^. In accordance with these characteristics, multiple tissue insults were seen in the brain, heart, and lungs in our asphyxia-induced CA rat model. Additionally, as consistent with our previous reports^6,7^, and further supported by this study with mRNA expression patterns, hyperoxia-induced oxidative stress injury after CA was considerable in the brain. In addition, we broadened the investigation into other pivotal organs such as the heart and lungs with an attempt to understand the tissue-specific patterns of oxidative stress post CA.

When a sudden CA occurs, circulation to the brain ceases and consciousness is lost within a second. Irreversible brain damage and death will rapidly follow if the sudden CA remains untreated. The chance of survival with a favorable neurological outcome declines rapidly the longer a patient remains in CA^11,31^. The same physiological insult to the brain is observed in our rat CA model with apparently decreased mNDS 24 h post CA following the elevation of circulating S100B concentrations, which has been reported as a sensitive marker of active neural distress^32^, in the acute phase after resuscitation. Likewise, the lungs are also susceptible to IR injury as well as hyperoxia-induced oxidative stress^8^. On the contrary, physiological insult to the heart was minimal in our asphyxia CA model as seen by negative findings of EF and FS by echocardiography at 24 h after CA. Although increased five-year risks of stroke, AF, ACS, and HF in survivors of OHCA^13^, and post-resuscitation myocardial dysfunction have been reported in both asphyxia CA and electrical fibrillation of the ventricle^33–35^, yet it has rarely been reported that asphyxia CA leads to severe myocardial dysfunction while potassium chloride-induced CA or electrical fibrillation of the ventricle does cause severer insults^33^. Potassium chloride-induced CA revealed that duration of CA prior to CPR determined a severity of myocardial dysfunction, though post-CPR cardiac dysfunction was not associated with myocardial inflammation, necrosis, or apoptosis, which is consistent with our findings in some extent^36^. Few reports have successfully shown the physiological insult of heart function along with detailed biological data such as mRNA expression. Our study focused on detailed analysis of these biological responses, showing that systemic IR injury increased inflammation related mRNA gene expression in the heart, yet it might not be associated with cardiac function or apoptotic signaling pathways.

We also measured troponin I concentrations in the plasma that showed significant increases after CA and resuscitation, however the concentration of 0.3–0.4 ng/ml were not as remarkable as those reported in myocardial infarction models^37,38^ or any other IR injuries to the heart tissue^39^, which generally shows at least 10 ng/ml after injury. This implies that our asphyxia-induced CA model might not cause severe myocardial injury but the troponin I is sensitive enough to identify a small insult that is caused by CA and resuscitation, including mechanical damage from chest compression.

Plasma concentrations of cardiac enzymes including troponin I may be affected by mechanical trauma due to chest compression during CPR. Lin CC et al. examined the influence of resuscitative procedures—defibrillation excluded—on the release of cardiac enzymes specifically from patients without any known confounding factors affecting cardiac enzyme release^40^. Their report revealed that troponin I concentration during 30 h after ROSC exhibited a bell-shaped configuration. This is distinct from that after AMI although the enzymatic activities of CKMB and CK were constantly higher than normal. These data suggest that chest compression during CPR may be associated with the little elevation of plasma troponin I concentrations as well as inflammation related mRNA gene expression like it was seen from our asphyxia CA model.

In the brain, relative mRNA gene expression of representative inflammatory signaling pathways (*Il1b, Il6, Tgfb1, Icam1, Nfkb1,* and *Traf6*) and representative apoptosis signaling pathways (*Casp3*, *Casp9*, *Egf*, and *Bax*) increased post CA, and these gene expression were significantly attenuated by normoxic therapy. Normoxic therapy significantly reduced oxidative stress-induced inflammatory and apoptosis related mRNA gene expression in the brain, whereas no statistical differences were found in the heart. Collectively, it is inferred that the increase of inflammation related mRNA gene expression in the heart might not be derived from hyperoxia-induced oxidative stress but might be due to resuscitation-induced heart injuries as described above. In addition, apoptosis related mRNA gene expression did not increase in the heart after CA, which is consistent with the tissue-specific phenotypic pattern of post-CA syndrome, in which the neurological dysfunction is indeed remarkable.

This study is subject to several limitations. Firstly, we studied bio-physiological insults post-CA in rats, the findings of which may not be fully applicable to human diseases^41^. Secondly, we quantified only superoxide as ROS generation, which is catalyzed by NOX, however, there are multiple enzymes associated with ROS generation, such as xanthine oxidase, and monoamine oxidase^42^. These enzymes are possible contributors to the oxidative stress that we found in our CA rats. Thirdly, bio-physiological insults were evaluated within 2 h post-resuscitation period and 24 h after CA in the present study, however it is possible that myocardial dysfunction can occur between or after these timings as myocardial stunning or injury in the early hours following CA with gradual recovery have been reported in some literature^36,43,44^. Finally, mRNA gene expression on the brain, heart, and lung tissue homogenates were addressed in this study, but other tissues such as the kidney, liver, and spleen are also worthy to attest biological responses post CA. Thus, further exploration is warranted.

## Conclusions

Systemic IR injury on the present asphyxia CA model might not have physiological insult to the heart as opposed to those observed in the brain and lungs. Upon systemic IR injury, concomitant hyperoxia-induced injury exacerbated inflammation/apoptosis signals in the brain and lungs but might not affect the heart as much, therefore, post-CA normoxic therapy might be more protective to the brain and lungs, but not to the heart.

## Supplementary Information

Supplementary Information 1.

Supplementary Information 2.

## Data Availability

The datasets used and/or analyzed during the current study are available from the corresponding author on reasonable request.

## Abbreviations

2-OH-E +: 2-Hydroxyethidium
8OHdG: 8-Hydroxy-2’-deoxyguanosine
BAX: B-cell leukemia/lymphoma-2 associated X protein
BCL2: B-cell leukemia/lymphoma-2
CA: Cardiac arrest
CASP3: Caspase-3
CASP9: Caspase-9
DAPI: 4, 6-Diamido-2-Phenylindole
DHE: Dihydroethidium
DPI: Diphenyleneiodonium chloride
EGF: Epidermal growth factor
EtCO2: End tidal carbon dioxide
FS: Fractional shortening
ICAM1: Intercellular adhesion molecule-1
IR: Ischemia–reperfusion
mNDS: Modified neurological deficit score
MV: Minute ventilation
NFKB1: Nuclear factor-kappa beta 1
OHCA: Out-of-hospital cardiac arrest
ROS: Reactive oxygen species
ROSC: Return of spontaneous circulation
TGFB1: Transforming growth factor-beta 1
TRAF6: Tumor necrosis factor receptor-associated factor-6
W/D: Wet/dry
