# Supplementary material for: Bio-physiological susceptibility of the brain, heart, and lungs to systemic ischemia reperfusion and hyperoxia-induced injury in post-cardiac arrest rats
Source: Sci Rep. 2023 Feb 28;13:3419. doi: 10.1038/s41598-023-30120-1 (PMC9974929; doi:10.1038/s41598-023-30120-1)
Supplement: Supplementary file 1 — Supplementary Information 1. [file 41598_2023_30120_MOESM1_ESM.pptx]

## Slide 1
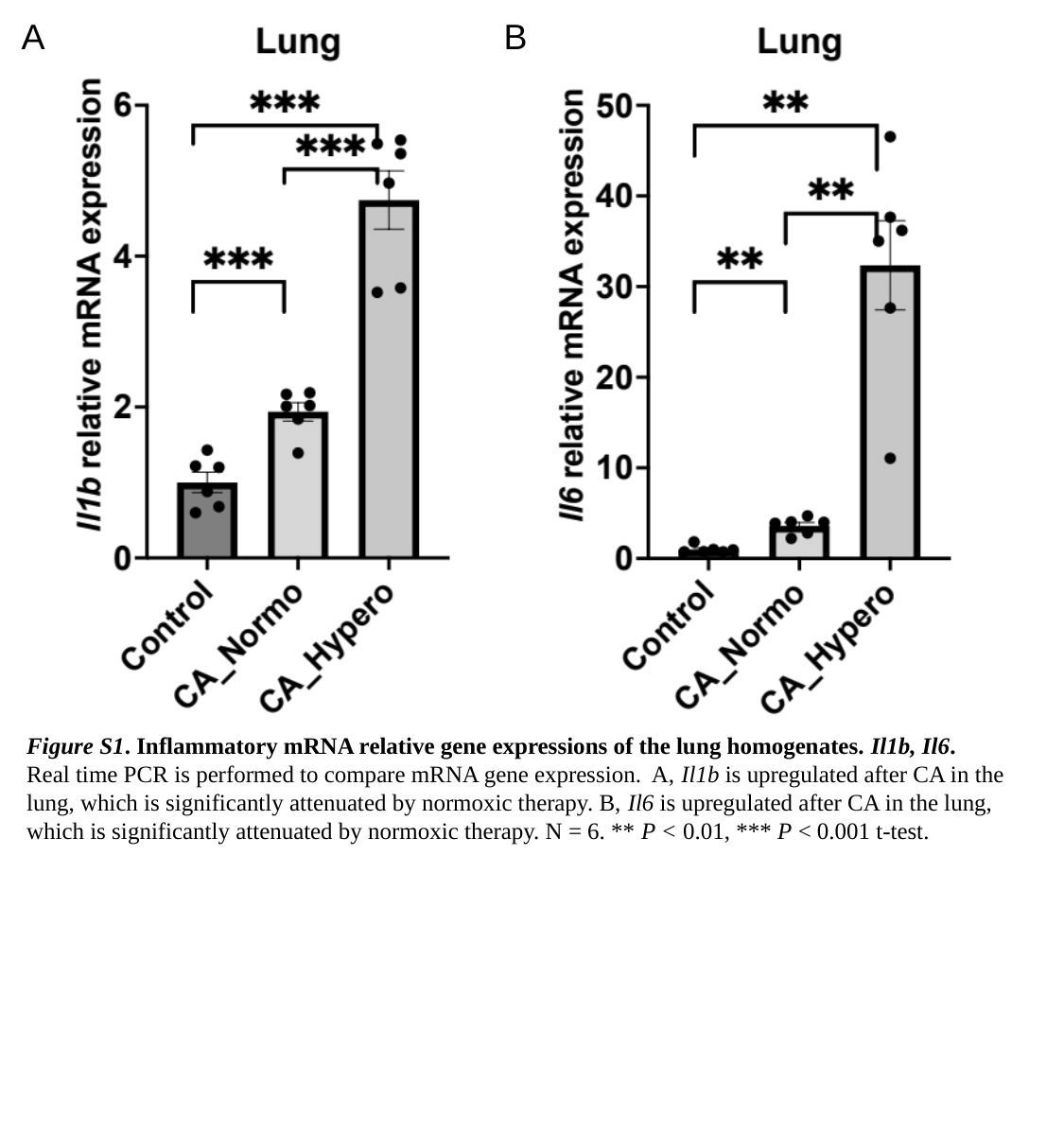

A
B
Figure S1. Inflammatory mRNA relative gene expressions of the lung homogenates. Il1b, Il6.
Real time PCR is performed to compare mRNA gene expression. A, Il1b is upregulated after CA in the lung, which is significantly attenuated by normoxic therapy. B, Il6 is upregulated after CA in the lung, which is significantly attenuated by normoxic therapy. N = 6. ** P < 0.01, *** P < 0.001 t-test.
