# Supplementary material for: Bio-physiological susceptibility of the brain, heart, and lungs to systemic ischemia reperfusion and hyperoxia-induced injury in post-cardiac arrest rats
Source: Sci Rep. 2023 Feb 28;13:3419. doi: 10.1038/s41598-023-30120-1 (PMC9974929; doi:10.1038/s41598-023-30120-1)
Supplement: Supplementary file 2 — Supplementary Information 2. [file 41598_2023_30120_MOESM2_ESM.docx]

**Supplemental Table S1.** Characteristics of the rats for organ damage assessment after cardiac arrest

| Variable | Baseline | 20-min  after CPR | *P* value  vs Baseline | 2-hour  after CPR | *P* value  vs Baseline |
| --- | --- | --- | --- | --- | --- |
| Body weight, g | 463.0 ± 14.7 |  |  |  |  |
| Surgical time, min | 34.1 ± 3.9 |  |  |  |  |
| Time to CA, sec | 211.9 ± 7.1 |  |  |  |  |
| Time to ROSC, sec | 64.4 ± 2.3 |  |  |  |  |
| ROSC rate | 100% (8/8) |  |  |  |  |
| 24-hour Survival rate | 87.5% (7/8) |  |  |  |  |
| Vital sign |  |  |  |  |  |
| MAP, mmHg | 87.2 ± 5.2 | 136.3 ± 4.5 | < 0.001*** | 109.2 ± 1.3 | 0.0037** |
| HR, bpm | 260.4 ± 14.3 | 384.9 ± 10.8 | < 0.001*** | 344.0 ± 15.8 | 0.0016** |
| RR, /min | 45.3 ± 0.2 | 45.2 ± 0.1 | 0.7710 | 45.3 ± 0.3 | 0.9126 |
| EtCO_2_, mmHg | 31.7 ± 1.5 | 42.3 ± 1.0 | < 0.001*** | 37.3 ± 1.8 | 0.0313* |
| Airway pressure, mmHg | 8.1 ± 0.2 | 9.7 ± 0.7 | 0.0414* | 10.3 ± 0.3 | < 0.001*** |
| Teso, ℃ | 36.6 ± 0.2 | 36.7 ± 0.04 | 0.5522 | 36.5 ± 0.1 | 0.6345 |

Data are presented as mean ± SEM.

CPR, cardiopulmonary resuscitation; CA, cardiac arrest; ROSC, return of spontaneous circulation; MAP, mean arterial pressure; HR, heart rate; RR, respiratory rate; EtCO_2_, end tidal carbon dioxide; Teso, esophageal temperature; SEM, standard error of measurement.

**P* < 0.05, ***P* < 0.01, ****P* < 0.001 in all variables
